# Antidepressant‐Like Effect of Ferulic Acid via Promotion of Energy Metabolism Activity

**DOI:** 10.1002/mnfr.201900327

**Published:** 2019-08-22

**Authors:** Kazunori Sasaki, Nozomu Iwata, Farhana Ferdousi, Hiroko Isoda

**Affiliations:** ^1^ Alliance for Research on the Mediterranean and North Africa (ARENA) University of Tsukuba 1‐1‐1 Tennodai Tsukuba Ibaraki 305–8572 Japan; ^2^ Interdisciplinary Research Center for Catalytic Chemistry National Institute of Advanced Industrial Science and Technology (AIST) AIST Tsukuba Central 5‐2 Tsukuba Ibaraki 305–8565 Japan; ^3^ Faculty of Pure and Applied Sciences University of Tsukuba 1‐1‐1 Tennodai Tsukuba Ibaraki 305–8571 Japan; ^4^ School of Integrative and Global Majors (SIGMA) University of Tsukuba 1‐1‐1 Tennodai Tsukuba Ibaraki 305–8577 Japan; ^5^ Faculty of Life and Environmental Sciences University of Tsukuba Japan1‐1‐1 Tennodai Tsukuba Ibaraki 305–8572 Japan

**Keywords:** Depression, dopaminergic synapse, energy metabolism, ferulic acid, tail suspension test

## Abstract

**Scope:**

Ferulic acid (FA), a natural phenolic phytochemical abundantly present in whole grains, herbs, and dried fruits, exhibits anti‐inflammatory, antioxidant, and neuroprotective effects. In the present study, the antidepressant‐like effects of FA in male ICR mice using tail suspension test (TST) are investigated and its molecular mechanisms are explored.

**Methods and Results:**

Oral administration of FA at a dose of 5 mg kg^–1^ for 7 days significantly reduces immobility of mice compared to vehicle‐administered control group. Microarray and real‐time PCR analyses reveal that FA upregulates the expression of several genes associated with cell survival and proliferation, energy metabolism, and dopamine synthesis in mice limbic system of brain. Interestingly, it is found that FA, unlike antidepressant drug bupropion, strongly promotes energy metabolism. Additionally, FA increases catecholamine (dopamine and noradrenaline), brain‐derived neurotrophic factor, and ATP levels, and decreases glycogen levels in the limbic system of the mice brain.

**Conclusion:**

The research provides the first evidence that FA enhances energy production, which can be the underlying mechanism of the antidepressant‐like effects of FA observed in this study.

## Graphical abstract

**Ferulic acid (FA)** enhances the energy metabolism of mice brain, which could be the underlying mechanism of the antidepressant‐like effects of FA in this study.

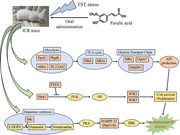

## 1 Introduction

Various sources of stress, such as mental, chemical, structural, and environmental are constantly increasing in modern societies. These types of stress, as well as chronic stress, may lead to major depression in humans. Depression is thought to be a major public health problem globally. Approximately 350 million people worldwide are affected by depression. It is expected to be ranked as the second‐leading cause of death by 2020.^1^ Common symptoms of depression include emotional disturbance, such as feelings of hopelessness and lack of motivation, and physical symptoms, such as sleep disturbance, malaise, and anorexia. Currently, several antidepressant drugs are available, such as monoamine oxidase inhibitors, selective serotonin reuptake inhibitors, selective norepinephrine inhibitors.^2^ However, a major limitation of these antidepressants is side effects such as vomiting, dysuria, and sexual dysfunction that cannot be ignored. It is, therefore, a great scientific interest to explore new effective, fast‐acting, and well‐tolerated drugs for the treatment of depression.

Recently, natural resources have received attention as effective alternatives in the treatment of depression. Natural resources are commonly regarded as safe‐to‐use because they have far fewer adverse effects than the synthetic antidepressant drugs currently in use.^3^ Therefore, it is preferable to use natural bioactive compounds such as polyphenols for the treatment of depression and other mood disorders. In our previous study, we evaluated the antidepressant‐like effect of *Cymbopogon schoenanthus* extract (CSE) in two predictive models of depression in mice; tail suspension test (TST) and forced swimming test. CSE treatment decreased immobility time of mice in both models. The effect of CSE on animal behavior was concordant with a significant reduction of corticosterone in serum and upregulation of neurotransmitters such as dopamine, adrenaline, and noradrenaline levels in the cerebral cortex of mice.^4^ We also analyzed active compounds in CSE using HPLC analysis, which identified several active compounds in CSE such as gallic acid, quercetin‐3‐rhamnoside, and ferulic acid (FA). In particular, FA is the most abundant polyphenol in CSE composing up to 10% of the total phenolic constituent.^4^

FA is a natural polyphenol (4‐hydroxy‐3‐methoxycinnamic acid), commonly found in food sources such as rice, wheat, barley, oranges, coffee, apples, and peanuts.^5^ In addition, FA is also categorized as a caffeoylquinic acid (CQA) derivative. Previously, we reported that 3,5‐diCQA^6^ and CQA derivatives from purple sweet potato extract^7^ had neuroprotective activities. Another CQA derivative rosmarinic acid (RA) showed antidepressant activity.^8^, ^9^ Therefore, FA, as a member of the family of CQA derivatives, is considered to have potential antidepressant‐like activity. In fact, Lenzi et al. has demonstrated that FA exerts antidepressant‐like effects through modulation of the antioxidant defense system.^10^ Another recent study has reported that the antidepressant‐like effects of FA observed in epileptic animal model were mediated by its anti‐inflammatory properties.^11^ However, the proposed mechanisms of antidepressant activity of FA may act in a complementary manner to exert acute changes necessary for antidepressant behavioral actions, and thus, are not sufficient to explain the molecular mechanism underlying its antidepressant‐like effects.

In the present study, we evaluated the antidepressant‐like effect of FA in ICR mice using TST, an animal model of depression. DNA microarray and real‐time PCR analyses were performed to examine the changes in gene expression in the limbic system of mice brain. Furthermore, the current research focuses on the modulation of catecholamine, corticosterone, and brain‐derived neurotrophic factor (BDNF), and energy metabolism for an understanding of the molecular mechanism underlying the antidepressant‐like effect of FA.

## 2 Experimental Section

### 2.1 Preparation of FA

FA was purchased from FUJIFILM Wako Pure Chemical Corporation. (Tokyo, Japan). FA was dissolved in 99.5% of methanol (stock solution of FA). The stock solution of FA was dissolved in drinking water and was orally administered at a concentration of 5 mg kg^–1^ body weight.

### 2.2 Animals and FA Administration in ICR Mice

Three‐week‐old male ICR mice (purchased from the Japan Charles River, Yokohama, Japan) were used for the in vivo experiment for evaluation of the antidepressant‐like activity. All mice were lodged individually and allowed to acclimate to the laboratory conditions for 7 days under controlled temperature (21–23 °C) and light conditions (light:dark 12:12 h) with free access to food and water. This animal experiment was approved by the Ethics Animal Care and Use Committee of the University of Tsukuba (16‐042).

Following acclimatization to laboratory conditions, mice were randomly divided into three groups: vehicle‐treated group (*n* = 7), bupropion‐treated group (*n* = 7), and FA‐treated group (*n* = 7). FA (5 mg kg^–1^) was mixed with drinking water and then directly administered by oral gavage with a feeding tube and syringe every day for 7 days. Previously, we performed several animal experiments using CQA derivatives such as 3,5‐diCQA,^6^ RA,^9^ and 3,4,5‐triCQA.^12^ These CQA derivatives showed neuronal activity at the following concentrations: 3,5‐diCQA at 6.7 mg kg^–1^; RA at 5 and 10 mg kg^–1^; and 3,4,5‐triCQA at 5 mg kg^–1^. Considering these previous studies, we set the concentration of FA to 5 mg kg^–1^. And final concentration of methanol in the FA‐mixed water was 0.1%. An equal volume of 0.1% methanol water was administered to the vehicle‐administration group. Bupropion (serotonin and noradrenaline reuptake inhibitor; DNRI; FUJIFILM Wako Pure Chemical Corporation. Tokyo, Japan) was used as a positive control. It was dissolved in distilled water and orally administered to mice at a volume of 20 mg kg^–1^ body weight, as reported in previous study.^9^

### 2.3 Tail Suspension Test

TST was performed 60 min after oral administration of FA as described previously.^9^ Briefly, the mice were held by the tail at 10 mm distance from the tip of the clamp (white box 30 × 15 × 50 cm (L × W × H)) with 30 cm headspace from the bottom of the box, which had minimal background noise. All mice were suspended for 6 min in each session and the immobility period was recorded in the last 4 min of the test. Mice were considered to be immobile only when they were passive and completely stationary. Immobility in the TST is assumed to reflect behavioral despair in clinical depression in humans.

### 2.4 RNA Isolation from Mouse Brain and DNA Microarray Analysis

After the last TST, all mice were sacrificed by cervical dislocation. The brain tissues were isolated, washed with ice‐cold PBS, and immediately immersed in liquid nitrogen. As previous studies have pointed out the functional and structural alterations in the limbic area, including cerebral cortex and amygdala, in mouse models of depression^9^ and in adult patients with major depressive disorder,^13^, ^14^ the limbic area of mice brain was focused to determine the mechanisms of anti‐depressant‐like effects of FA. The entire limbic area of the mouse brain containing the cortex, hippocampus, and amygdale was quickly dissected on ice and was minced for RNA extraction. Total RNA was purified from cerebral tissue using the ISOGEN kit (Nippon Gene Co. Ltd., Tokyo, Japan) following the manufacturer's instructions as reported previously.^15^ Total RNA was quantified and assessed for quality with NanoDrop 2000 spectrophotometer (Thermo SCIENTIFIC, Wilmington, DE, USA).

DNA microarray analysis was performed as reported previously.^15^ Double‐stranded cDNA was synthesized from 100 ng of total RNA with the GeneAtlas 3′ IVT Express Kit (Affymetrix Inc., Santa Clara, CA, USA). Biotin‐labeled amplified RNA (aRNA) was synthesized by transcription using the GeneChip 3′ IVT Express Kit (Affymetrix Inc., Santa Clara, CA, USA). Purified aRNA (9.4 µg) was fragmented using the GeneAtlas 3′ IVT Express Kit and was hybridized for 16 h at 45 °C using GeneChip MG‐430 PM microarray (Affymetrix Inc., Santa Clara, CA, USA). The chip was washed and stained in the Gene Atlas Fluidics Station 400 (Affymetrix Inc., Santa Clara, CA, USA) and the resulting image was scanned using the GeneAtlas Imaging Station (Affymetrix Inc., Santa Clara, CA, USA). The raw data was normalized using the Affymetrix expression console (http://www.affymetrix.com). Subsequent analysis of the gene expression data was carried out using an online data mining tool DAVID (Database for Annotation, Visualization and Integrated Discovery, v6.8, National Institute of Allergy and Infectious Diseases). Compared with the control (vehicle‐treated group), the fold‐change in gene expression in the bupropion‐ or FA‐treated group was calculated and converted to linear data.

### 2.5 Real‐Time PCR

On the basis of findings from microarray analysis, reverse‐transcription reactions were carried out with the SuperScript III Reverse Transcriptase kit (Invitrogen, Carlsbad, CA, USA) according to the manufacturer's instructions. One microgram of total RNA template solution, 10 mm dNTP (Invitrogen, Carlsbad, CA, USA), Oligo (dT)_12‐18_ primer (Invitrogen, Carlsbad, CA, USA), and UltraPure DNase/RNase‐Free Distilled Water (Gibco, USA) were mixed and incubated at 65 °C for 5 min and chilled at 4 °C for 1 min. After adding 5× Reverse Transcriptase buffer, 0.1 m DTT, Reverse Transcriptase, and RNaseOUT (Invitrogen, Carlsbad, CA, USA), the solution was incubated at 42 °C for 60 min, then at 70 °C for 10 min. The synthesized complementary DNA (cDNA) solution was quantified using the Nanodrop 2000 spectrophotometer (Thermo SCIENTIFIC, Wilmington, DE, USA) and stored at –20 °C until use. For the transcript quantification, the TaqMan real‐time RT‐PCR amplification reactions were performed using the Applied Biosystems 7500 Fast Real‐Time System (Applied Biosystems, Foster City, CA, USA). All primers and the TaqMan Universal PCR Master Mix were obtained from Applied Biosystems. Specific primers for beta actin (*Actb*) (Mm02619580_s1), hexokinase (*Hk*) (Mm02526975_g1), glyoxalase 1 (*Glo1*) (Mm00844954_s1), pyruvate kinase (*Pk*) (Mm00834102_gH), 3‐phosphoglycerate dehydrogenase (*Phgdh*) (Mm01623589_g1), pyruvate dehydrogenase kinase, isoenzyme 4 (*Pdk4*) (Mm01166879_m1), pyruvate carboxylase (*Pc*) (Mm00500992_m1), tyrosine hydroxylase (*Th*) (Mm004427557_m1), and dopa decarboxylase (*Ddc*) (Mm00516688_m1) were used. Amplification was performed in a final volume of 20 µL using 9 µL of cDNA solution (containing 100 ng of cDNA), 1 µL of each corresponding primer, and 10 µL of TaqMan Gene Expression Master Mix. The thermal cycler program was set as 50 °C for 2 min and 95 °C for 10 min, followed by 45 cycles of PCR (95 °C, 15 s; 60 °C, 60 s). Results were obtained from three independent experiments, and the mRNA levels of all genes were normalized using *Actb* as an internal control.

### 2.6 Dopamine and Noradrenaline Levels in Mice Brain

Dopamine and noradrenaline levels in the limbic area of mice brains were measured using a commercial ELISA kit based on the sandwich principle (ImmuSmol, inc., Pessac, France). Briefly, 100 mg of the limbic area was isolated from brain tissue and homogenized in radioimmunoprecipitation assay (RIPA) buffer with protease inhibitor (Santa Cruz Biotechnology, inc., Tokyo, Japan). After centrifugation (1000 × *g*, 20 min), 50 µL supernatant or 10 µL standards were used for catecholamine extraction, acylation, and determination following manufacturer's instructions. After optimal color development, the reaction was stopped and the absorbance was recorded at 450 nm. Dopamine and noradrenaline levels were computed by correcting the protein concentration. Protein concentration was determined using the 2D Quant kit (GE Healthcare Inc., Tokyo, Japan) and the data were expressed as ng µg^–1^ protein.

### 2.7 BDNF Level in Mice Brain

BDNF levels in the brain were measured by ELISA kit (R&D Systems, Inc., Minneapolis, USA) in accordance with the manufacturer's instructions. Briefly, 100 mg of the limbic area was isolated from brain tissue and homogenized in RIPA buffer with protease inhibitor (Santa Cruz Biotechnology, Inc., Tokyo, Japan). After centrifugation (1000 × *g*, 20 min), the supernatants or standards were used to determine the BNDF levels in brain. After the treatment of BNDF antibody, a second incubation was performed with streptavidin–horseradish peroxidase conjugate solution for 60 min. After addition of substrate and stop solution, BDNF levels were determined by absorbance at 450 nm. The BDNF levels were normalized to the protein concentration determined with the 2D Quant kit (GE Healthcare Inc.). The data were expressed as ng µg^–1^ protein.

### 2.8 Measurement of Brain Glycogen Levels

Biochemical quantification of glycogen was performed using commercial glycogen assay kit (BioVision Inc., Milpitas USA). After homogenization of the limbic area of mice brain, the homogenates were centrifuged (1000 × *g*, 20 min) and the supernatant was collected. The supernatant of samples or glycogen standards were transferred to a 96‐well plate, followed by incubation with 1 µL hydrolysis enzyme mix for 30 min. The samples were incubated with 1 µL development enzyme mix and 0.3 µL OxiRed probe for 30 min at room temperature. The absorbance of samples was measured by a microplate reader (405 nm). The glycogen concentration was then calculated based on a calibration curve obtained by the glycogen standard. Protein concentration was estimated using the 2D Quant kit (GE Healthcare Inc.) and the data were expressed as ng µg^–1^ protein.

### 2.9 Measurement of Brain ATP Levels

ATP levels in the cerebrum tissues were measured using a commercial glycogen assay kit (BioVision Inc., Milpitas, USA). A small amount (100 mg) of the limbic area of the brain was homogenized with 1 mL RIPA buffer with protease inhibitor (Santa Cruz Biotechnology, Inc., Tokyo, Japan). After centrifugation (1000 × *g*, 20 min), the supernatant or ATP standard was transferred to a 96‐well plate. The samples were incubated with 1 µL development enzyme mix and 0.3 µL OxiRed probe for 30 min at room temperature. The absorbance of each sample was measured by a microplate reader (405 nm). Glycogen concentration was then calculated based on the calibration curve obtained by the ATP standard. Protein concentration was estimated using the 2D Quant kit (GE Healthcare Inc.) and the data were expressed as ng µg^–1^ protein.

### 2.10 Corticosterone Level in Mice Serum

Corticosterone levels in the serum were measured using Corticosterone ELISA kit (Proteintech Inc., Tokyo, Japan). After 24 h of incubation at 4 °C, the blood was centrifuged at 1000 × *g* for 30 min to collect the serum and was immediately stored at −20 °C until use. Serum samples or standards (10 µL) were used for corticosterone determination according to manufacturer's instructions. When optimal blue color density developed, the reaction was stopped and the absorbance was recorded at 450 nm. The level of serum corticosterone was calculated using a standard curve. All samples were subjected to single assay and the results were expressed in ng mL^–1^.

### 2.11 Statistical Analysis

Results are expressed as mean ± SEM. Immobility time in TST was compared between control and treatment groups using two‐way ANOVA followed by post hoc Ryan–Einot–Gabriel–Welsch multiple range test. One‐way ANOVA followed by Ryan–Einot–Gabriel–Welsch multiple range test was also carried out. A *p*‐value of <0.05 was considered statistically significant.

## 3 Results

### 3.1 FA Reverses depressive‐like Behavior Induced by TST

**Figure 1:**
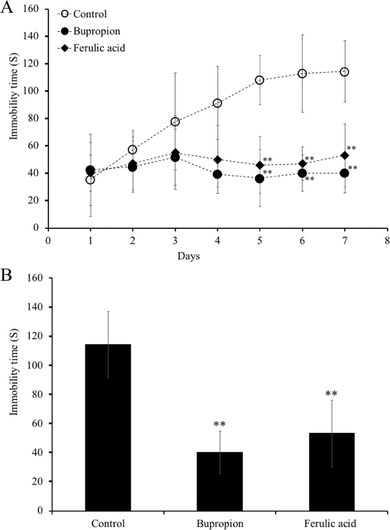
Effects of administration of ferulic acid (FA) on immobility times in the tail suspension test (TST). Mice were orally‐administered daily with vehicle (0.1% methanol water), bupropion (20 mg kg^–1^), or FA (5 mg kg^–1^) for 7 consecutive days and subjected to TST 60 min after sample administration. The immobility time during the final 4 min of a 6‐min total session was measured. TST immobility time measured for each group. Data represent the mean ± SEM (*n* = 7). **p < 0.01 versus control group.

To determine whether FA has antidepressant‐like activity, its effect on TST‐induced stress in mice was investigated. We found that vehicle‐treated group displayed a significant increase in immobility time in TST (35.5 ± 26.9, 57.4 ± 13.7, 77.8 ± 35.3, 91.5 ± 26.7, 108.1 ± 18.0, 112.9 ± 28.4, and 114.4 ± 22.5 s, respectively; **Figure** 1A). On the other hand, FA administration (5 mg kg^–1^) caused a decrease in the immobility time in the TST (40.1 ± 13.3, 47.1 ± 19.3, 55.0 ± 27.1, 50.0 ± 24.8, 46.0 ± 20.8, 46.9 ± 12.1, and 53.2 ± 23.1 s, respectively). The immobility times of the bupropion‐treated group (20 mg kg^–1^), as a positive control, also showed a decrease (42.5 ± 25.9, 44.7 ± 18.7, 51.8 ± 20.4, 39.4 ± 9.4, 36.4 ± 20.8, 40.1 ± 13.1, and 40.2 ± 14.7 s, respectively) during TST.

Interestingly, on day 7, the FA‐administered group (53.2 ± 23.1 s) showed almost the same decrease in immobility time as the bupropion‐administered group (40.2 ± 14.7 s). Treatment with FA and bupropion resulted in twofold reduction of the average immobility time compared with that of the vehicle‐administered group (*p* < 0.01 vs control; Figure 1B).

### 3.2 FA Upregulated Energy Metabolism‐, Cell Proliferation‐, and Dopaminergic Synthesis‐Related Signaling Pathway Genes

To evaluate the molecular mechanism of the antidepressant‐like effect of FA, we performed DNA microarray analysis of ICR mice cerebrum to investigate changes in gene expression.

As shown in Table 1, 16 genes were upregulated in the FA‐treated group to the vehicle‐treated group. These genes were functionally classified into three groups: energy metabolism, cell survival and proliferation, and dopamine synthesis (Table 1).

More specifically, FA treatment induced the expression of energy metabolism signaling‐related genes, namely glyoxalase 1 (*Glo1*), aldolase C, fructose‐bisphosphate (*Aldoc*), pyruvate kinase (*Pk*), phosphoglucomutase 2 (*Pgm2*), 3‐phosphoglycerate dehydrogenase (*Phgdh*), isocitrate dehydrogenase 3 (NAD+) alpha (*Idh3a*), pyruvate dehydrogenase kinase, isoenzyme 4 (*Pdk4*), succinate dehydrogenase complex, subunit A (*Sdha*), ubiquinol‐cytochrome c reductase core protein 1 (Uqcrc1), and NADH dehydrogenase (ubiquinone) 1 alpha/beta subcomplex 1 (*Ndufab1*). FA also increased several gene expressions related to cell survival and proliferation signaling such as IFN (α and β) receptor 1 (*Ifnar1*), serum/glucocorticoid regulated kinase 3 (*Sgk3*), phosphatase and tensin homolog (*Pten*), and serum/glucocorticoid regulated kinase 1 (*Sgk1*). Moreover, FA increased the expression of several genes related to dopaminergic synthesis signaling pathways such as dopa decarboxylase (*Ddc*) and protein phosphatase 1, regulatory (inhibitor) subunit 1B (*Ppp1r1b*). However, the bupropion‐treated mice did not upregulate the genes related to energy metabolism.

**Table 1:** Expression changes of cell survival and proliferation‐, energy metabolism‐, dopamine synthesis‐related genes regulated by FA

Gene title	Gene symbol	Bupropion	Ferulic acid	Function
IFN (α and β) receptor 1	*Ifnar1*	**1.07**	**1.20**	Cell survival and proliferation
Serum/glucocorticoid regulated kinase 3	*Sgk3*	**1.03**	**1.21**	
Phosphatase and tensin homolog	*Pten*	**1.21**	**1.51**	
Serum/glucocorticoid regulated kinase 1	*Sgk1*	**1.04**	**1.53**	
Glyoxalase 1	*Glo1*	**0.89**	**1.43**	Energy metabolism (glycolysis)
Aldolase c, fructose‐bisphosphate	*Aldoc*	**1.13**	**1.35**	
Pyruvate kinase	*Pk*	**1.12**	**1.37**	
Phosphoglucomutase	*Pgm*	**0.98**	**1.21**	
3‐Phosphoglycerate dehydrogenase	*Phgdh*	**1.06**	**1.42**	
Isocitrate dehydrogenase 3 (NAD+) alpha	*Idh3a*	**1.18**	**1.23**	Energy metabolism (TCA cycle)
Pyruvate dehydrogenase kinase, isoenzyme 4	*Pdk4*	**1.18**	**1.33**	
Succinate dehydrogenase complex, subunit A, flavoprotein (Fp)	*Sdha*	**1.05**	**1.21**	Energy metabolism (electron transport chain)
Ubiquinol‐cytochrome c reductase core protein 1	*Uqcrc1*	**1.06**	**1.21**	
NADH dehydrogenase (ubiquinone) 1, alpha/beta subcomplex, 1	*Ndufab1*	**1.10**	**1.27**	
Dopa decarboxylase	*Ddc*	**0.52**	**1.30**	Dopamine synthesis
Protein phosphatase 1, regulatory (inhibitor) subunit 1B	*Ppp1r1b*	**2.43**	**1.59**	

### 3.3 Effects of FA on the Gene Expression of Energy Metabolism‐ and Dopamine Synthesis‐Related Mediators

**Figure 2:**
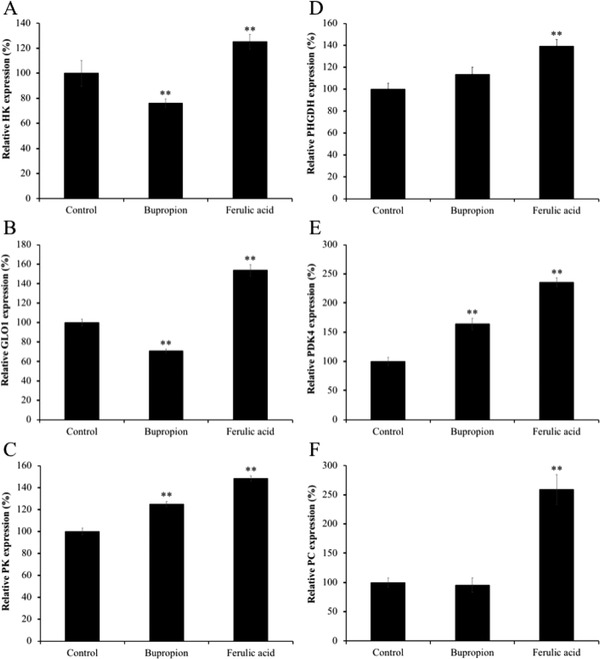
Effect of the administration of ferulic acid (FA) on mRNA expression of energy metabolism‐related genes in mice brain limbic system. Gene expression level of A) *Hk*, B) *Glo1*, C) *Pk*, D) *Phgdh*, E) *Pdk4*, and F) *Pc*, and were normalized to *Actb* level and expressed as a ratio relative to the control group. Each bar represents the mean ± SEM (*n* = 7 independent experiments). *p < 0.05; **p < 0.01 treatment versus control group.

**Figure 3:**
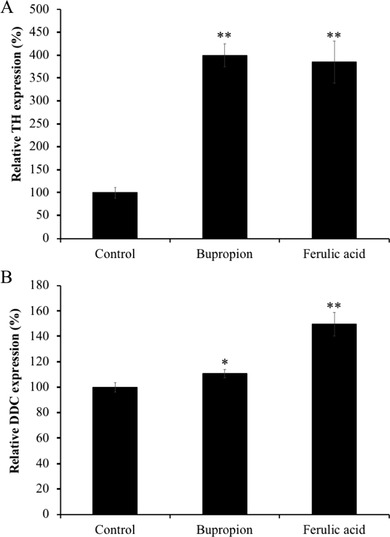
Effect of the administration of ferulic acid (FA) on mRNA expression of dopamine synthesis‐related genes in mice brain limbic system. Gene expression level of A) *Th* and B) *Ddc* were normalized to *Actb* level and expressed as a ratio relative to the control group. Each bar represents the mean ± SEM (*n* = 7 independent experiments). *p < 0.05, **p < 0.01 treatment versus control group.

As our microarray results indicated, FA affected the energy metabolism‐, cell survival and proliferation‐, and dopamine synthesis‐related genes expression in mouse brain. Especially, upregulation of energy metabolism‐related genes was characteristic in the FA‐administrated mice. Therefore, the gene expression levels of *Glo1*, *Pk*, *Phgdh*, and *Pdk4*, which are related to energy metabolism, were further evaluated in the brains of FA‐administrated mice. Oral administration with FA significantly increased gene expression levels of *Glo1*, *Pk, Phgdh*, and *Pdk4* up to 154.0%, 148.4%, 139.3%, and 235.9%, respectively (**Figure** 2). In addition, we also evaluated gene expression levels of hexokinase (*Hk*), rate‐limiting enzymes of the glycolysis and pyruvate carboxylase (*Pc*), and tricarboxylic acid (TCA) cycle. We found upregulation of *Hk* and *Pc* by 125.3% and 259.3%, respectively (Figure 2). Moreover, tyrosine hydroxylase (*Th*), the rate‐limiting enzyme in the dopamine synthesis, and *Ddc*, dopamine synthesis‐related genes, were significantly increased to 385.1% and 149.9% in mouse brains treated with FA (**Figure** 3).

Bupropion treatment also significantly upregulated (*p* < 0.01) *Th* and *Ddc* expression by 400.0% and 110.9% compared to the control group (Figure 3). However, the gene expressions of *Glo1*, *Pk*, *Phgdh*, *Pdk4, Hk*, and *Pc* were much lesser in the bupropion‐treated group compared to those of in the FA‐treated group (70.8%, 125.1%, 113.4%, 164.2%, 76.2%, and 95.8%, respectively; Figure 2).

### 3.4 Effects of FA on Dopamine, Noradrenaline, and BDNF Levels in the Limbic System of Mice Brain

**Figure 4:**
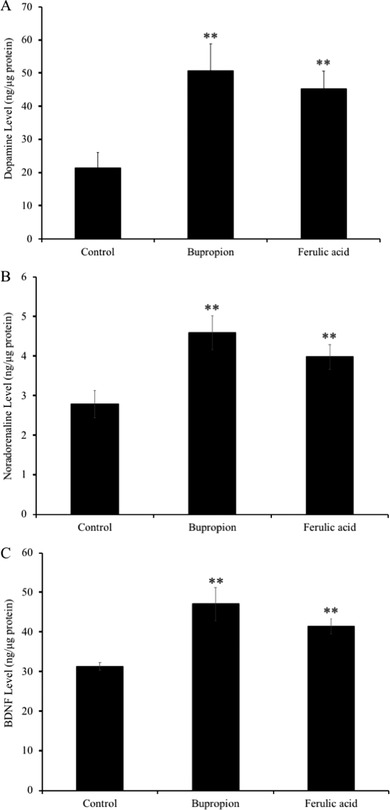
Effect of administration of ferulic acid (FA) on the levels of the A) dopamine, B) noradrenaline, and C) brain‐derived neurotrophic factor (BDNF) in mice brain limbic system. Mice were administered orally with vehicle (0.1% methanol water), bupropion (20 mg kg^–1^ d^–1^), or FA (5 mg kg^–1^ d^–1^) for 7 days and subjected to TST 60 min after drug administration. Dopamine, noradrenaline, and BDNF in the brain limbic system were quantified using ELISA as detailed in Experimental Section. Each bar represents the mean ± SD (*n* = 7). **p < 0.01 treatment versus control group.

As shown in **Figure** 4, the oral administration of FA significantly elevated the contents of dopamine (45.3 ± 5.4 ng µg^–1^ protein), noradrenaline (4.0 ± 0.3 ng µg^–1^ protein), and BDNF (41.5 ± 1.9 ng µg^–1^ protein) levels in the limbic system of the mice brain compared with those of in the vehicle‐treated control group (21.4 ± 4.7 ng µg^–1^ protein, 2.8 ± 0.3 ng µg^–1^ protein, 31.3 ± 1.0 ng µg^–1^ protein, respectively; *p* < 0.01). The bupropion‐treated group also showed significantly increased dopamine (50.8 ± 8.1 ng µg^–1^ protein), noradrenaline (4.6 ± 0.4 ng µg^–1^ protein), and BDNF (47.0 ± 4.2 ng µg^–1^ protein) levels in the mice brain compared with those of in the vehicle‐treated group (*p* < 0.01). However, there was no significant difference in these parameters between FA‐and bupropion‐treated groups.

### 3.5 Effects of FA on Glycogen and ATP Levels in the Limbic System of Mice

**Figure 5:**
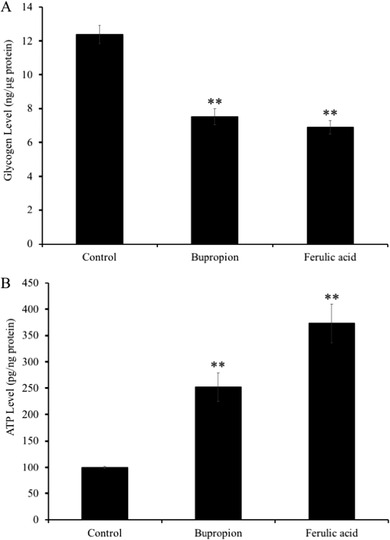
Effect of administration of ferulic acid (FA) on the levels of A) the glycogen and B) ATP in mice brain limbic system. Mice were administered orally with vehicle (0.1% methanol water), bupropion (20 mg kg^–1^ d^–1^), or FA (5 mg kg^–1^ d^–1^) for 7 days and subjected to TST 60 min after drug administration. Glycogen and ATP in the brain limbic system were quantified using a colorimetric assay kit as detailed in Materials and Method. Each bar represents the mean ± SD (*n* = 7). **p < 0.01 treatment versus control group.

Brain glycogen level was significantly decreased (*p* < 0.01) in FA‐treated group (6.9 ± 0.4 ng µg^–1^ protein) compared with that of in vehicle‐treated group (12.4 ± 0.5 ng µg^–1^ protein). Bupropion treatment also significantly decreased brain glycogen level (6.9 ± 0.4 ng µg^–1^ protein) compared with control group (*p* < 0.01; **Figure** 5A).

Similarly, ATP level in the FA‐administered (373.7 ± 36.9 pg µg^–1^ protein) and bupropion‐administered (252.2 ± 27.3 pg µg^–1^ protein) groups was increased significantly (*p* < 0.01) compared with that of in the vehicle‐administered group (100.3 ± 2.2 pg µg^–1^ protein). Interestingly, the changes in glycogen and ATP levels were much higher in the FA‐administered group compared to those of in bupropion‐administered group.

### 3.6 Effects of FA on Serum Corticosterone Level in Mice Blood Serum

**Figure 6:**
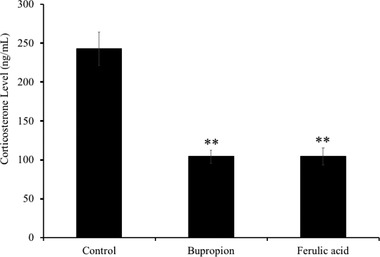
Effects of administration of ferulic acid (FA) on serum corticosterone levels in the mice subjected to TST. Mice were administered orally with vehicle (0.1% methanol water), bupropion (20 mg kg^–1^ d^–1^), or FA (5 mg kg^–1^ d^–1^) for 7 days and subjected to TST 60 min after drug administration. Corticosterone levels in mice blood serum were quantified using ELISA as indicated in Experimental Section. Each bar represents the mean ± SD (*n* = 7). **p < 0.01 treatment versus control group.

As shown in **Figure** 6, FA‐administered group elicited a significant reduction (*p* < 0.01) in serum corticosterone level in mice (105.1 ± 10.9 ng mL^–1^) compared to vehicle‐administered group (243.1 ± 21.6 ng mL^–1^). Similar effect was observed in the bupropion‐administered group (104.4 ± 8.5 ng mL^–1^, P < 0.01 vs control). Both FA and bupropion showed similar reduction in the TST‐induced increased level of serum corticosterone.

## 4 Discussion

The rapidly growing functional food industry may find promising remedies to overcome this problem. In the present study, we investigated the antidepressant‐like effects of FA through an acute mouse behavioral model of despair task TST. The TST has been widely used as an animal model to evaluate the pharmacological antidepressant activity.^16^ The present study shows that FA, when administered orally at the dose of 5 mg kg^–1^ body weight, effectively reduced immobility time in the TST‐subjected mice. Moreover, FA treatment significantly decreased serum corticosterone levels, in a similar way to bupropion, which is a commercial anti‐depressant drug. Also, our study demonstrated that FA significantly increased the protein‐expression level of BDNF in mice brain, the predictive biomarker of depression. All together the decrease of the immobility time in the TST,^17^ the decrease of serum corticosterone levels,^9^ and the increase of brain BDNF levels^18^ can be considered as an index for antidepressant effect, suggesting the antidepressant‐like effect of FAs.

Previous studies reported early dysregulation of mitochondrial functions as a symptom of major depression in adult patients.^19^ Previous evidences showed that mitochondrial function and energy metabolism are important for social behavior.^20^ In our microarray results, we reported an increase in the expression of *Glo1, Aldoc, Pk, Pgm*, and *Phgdh* genes, which are all involved in glycolysis (Table 1). Moreover, we also confirmed the upregulation of *IDH3a*, *Pdk4*, *Sdha*, *Uqcrc1*, and *Ndufab* in mice brains treated with FA, which are related to the TCA cycle and electron transport chain in mitochondria (Table 1). In addition, FA treatment significantly increased the gene expression levels of *Hk* and *Pc*, the rate‐limiting enzymes of the glycolysis and TCA cycle. It was previously reported that there were significant loss of mitochondrial ATP production and reduction in mitochondrial enzyme ratio in the patients with major depression.^21^ This present study shows that FA treatment increased the ATP level in the limbic area of the brain of ICR mice. Therefore, these results suggest that the ATP production in FA‐treated cells was increased due to activation of genes related to energy metabolism. Despite the implication of glucocorticoids in glucose metabolism disorder and in comorbidity between depression and diabetes, only a few studies have attempted to determine the role of glycolytic energy metabolism in any model of depression.^22^ Thus, our study provides the first evidence of antidepressant effects via promotion of energy metabolism by FA.

Glycogen, a complex glucose polymer found in the brain and liver, is usually considered as an energy store. Although brain glycogen is crucial for sustaining neuronal function under conditions of high energetic challenge such as hypoxia or hypoglycemia,^23^, ^24^ glycogen metabolism is profoundly affected by stress. For example, sleep deprivation decreased the glycogen level in the rat hippocampus,^25^ and surgical stress resulted in the reduction in brain glycogen.^26^ In the present study, we found that administration of bupropion or FA significantly decreased brain glycogen levels compared with TST‐stressed untreated mice (vehicle‐administered mice). Unlike brain glycogen levels, however, brain ATP levels in bupropion‐ or FA‐administered mice were significantly increased compared with that of in the TST‐stressed mice (vehicle‐administrated mice). Moreover, our results of microarray and real‐time PCR showed FA treatment upregulated the expressions of genes related to energy metabolism pathways such as glycolysis and TCA cycle. These results suggested that FA treatment increased brain ATP levels by promoting brain glycogen metabolism. Generally, ATP is essential for neuronal activities such as proliferation and differentiation. Therefore, promotion of brain ATP levels by FA‐administration is considered to contribute to the activation of neural functions.

On the other hand, our microarray results showed that FA upregulated of the gene expression of *Ddc* and *Ppp1r1b*, which are involved in dopaminergic signaling. We also found that FA upregulated the *Th* gene expression in mice brains. The monoamine hypothesis suggested that depression may be the result of dysregulation of monoaminergic neurotransmitters such as dopamine in the central nervous system. Dopamine is one of the most abundant monoamine neurotransmitters in the brain and plays an important role in regulating emotion, motivation, and cognition.^27^, ^28^ *Ddc* encoding the enzyme aromatic 1‐amino acid decarboxylase, an essential enzyme in various neurotransmitter pathways, is an interesting candidate gene for mental illness and psychiatric disorders.^29^ It has also been reported that the enzyme encoded by the *Ddc* gene is required for the synthesis of dopamine, norepinephrine, and serotonin.^30^ *Ppp1r1b*, which encodes dopamine and cAMP regulatory neuron phosphoprotein, is involved in the regulation of dopaminergic and glutamatergic signaling and in various neurological and psychological disorders including schizophrenia and depression.^31^, ^32^ Moreover, *Th* is the rate‐limiting enzyme in dopamine synthesis and is active in its phosphorylated form.^33^ Activated *Th* stimulates the production of dopamine, so *Th* levels can be used as a marker for the dopamine production.^34^, ^35^ Results obtained from our study showed that FA treatment increased brain dopamine and noradrenaline levels suggesting the promoting effect of FA in dopamine production.

In our previous study, we reported that CQA derivatives promoted energy metabolism through upregulating the genes associated with glycolysis,^6^ TCA cycle,^7^ and intracellular ATP production.^6^, ^36^ Moreover, metabolomics analysis showed that 3,5‐diCQA and 3,4,5‐triCQA exhibited promotion of central metabolic pathways such as glycolysis and the TCA cycle.^37^ Interestingly, FA, one of the CQA derivatives, also showed upregulation of genes related to energy production and induced the decrease of glycogen and the increase of ATP levels in the limbic area of mice brain in our present work. There are only few publications to date in PubMed that reported impaired energy metabolism in major depressive disorder.^38^, ^39^, ^40^, ^41^, ^42^, ^43^, ^44^ Therefore, our current research on the promotion of energy metabolism by FA may contribute to a new strategy toward a therapeutics for the treatment of depression.

Taken together, our present research suggests that FA exerts antidepressant‐like effects in an animal model of depression. Such properties appear to be mediated by enhancement of cell survival and proliferation, energy metabolism, and dopamine synthesis in mouse brain. Notably, the energy metabolism‐promoting effects of FA were not observed in the bupropion‐treated group that was used as positive control in this study. These newly discovered effects of FA may contribute to new strategies for the treatment of neurodegeneration‐induced depression.

## Conflict of Interest

The authors declare no conflict of interest.
